# Exploring correlations: Human seminal plasma and blood serum biochemistry in relation to semen quality

**DOI:** 10.1371/journal.pone.0305861

**Published:** 2024-06-24

**Authors:** Sylwia Płaczkowska, Kamil Rodak, Agnieszka Kmieciak, Iwona Gilowska, Ewa Maria Kratz

**Affiliations:** 1 Teaching and Research Diagnostic Laboratory, Department of Laboratory Diagnostics, Wroclaw Medical University, Wroclaw, Poland; 2 Division of Laboratory Diagnostics, Department of Laboratory Diagnostics, Wroclaw Medical University, Wroclaw, Poland; 3 Institute of Health Sciences, University of Opole, Opole, Poland; 4 Clinical Center of Gynecology, Obstetrics and Neonatology in Opole, Reference Center for the Diagnosis and Treatment of Infertility, Opole, Poland; University of Hyderabad, INDIA

## Abstract

Male infertility is a pressing global issue, prompting the need for biomarkers correlating with seminal parameters for diagnosis. Our study investigated 10 biochemical and energetic parameters in the seminal plasma and blood sera of fertile (25 subjects) and infertile (88 subjects) Polish men, correlations between their levels in seminal plasma and semen quality, and correlations between blood sera and seminal plasma levels of examined parameters. Infertile men displayed elevated seminal plasma glucose and fructose but reduced HDL levels compared to fertile men. We observed also weak negative correlations between seminal plasma triglycerides and sperm concentration in both groups. Moreover, infertile men exhibited positive correlations between seminal plasma HDL/LDL concentrations and sperm concentration. Fertile men showed moderate negative correlations between glucose/triglycerides concentrations and sperm count and between seminal plasma triglycerides levels and sperm vitality. Semen volume correlated with triglycerides (negative) and fructose (positive) concentrations in infertile men. Sperm motility correlated negatively with total cholesterol, LDL, and triglycerides concentrations in fertile men, and weakly with AMP-activated protein kinase in infertile men. Weak negative correlations between seminal plasma fructose/AMP-activated protein kinase concentrations and sperm progressive motility were observed in infertile men, whereas in fertile men seminal plasma AMP-activated protein kinase levels were positively correlated with progressive motility. Correlation analysis between blood serum and seminal plasma parameters revealed intriguing connections, notably regarding LDL, AMP-activated protein kinase, and carnitine, suggesting systemic influences on seminal plasma composition. These findings emphasize the complex interplay between metabolic factors and sperm parameters, offering promising directions for future research in male infertility diagnostics and therapeutics.

## Introduction

**Fig 1:**
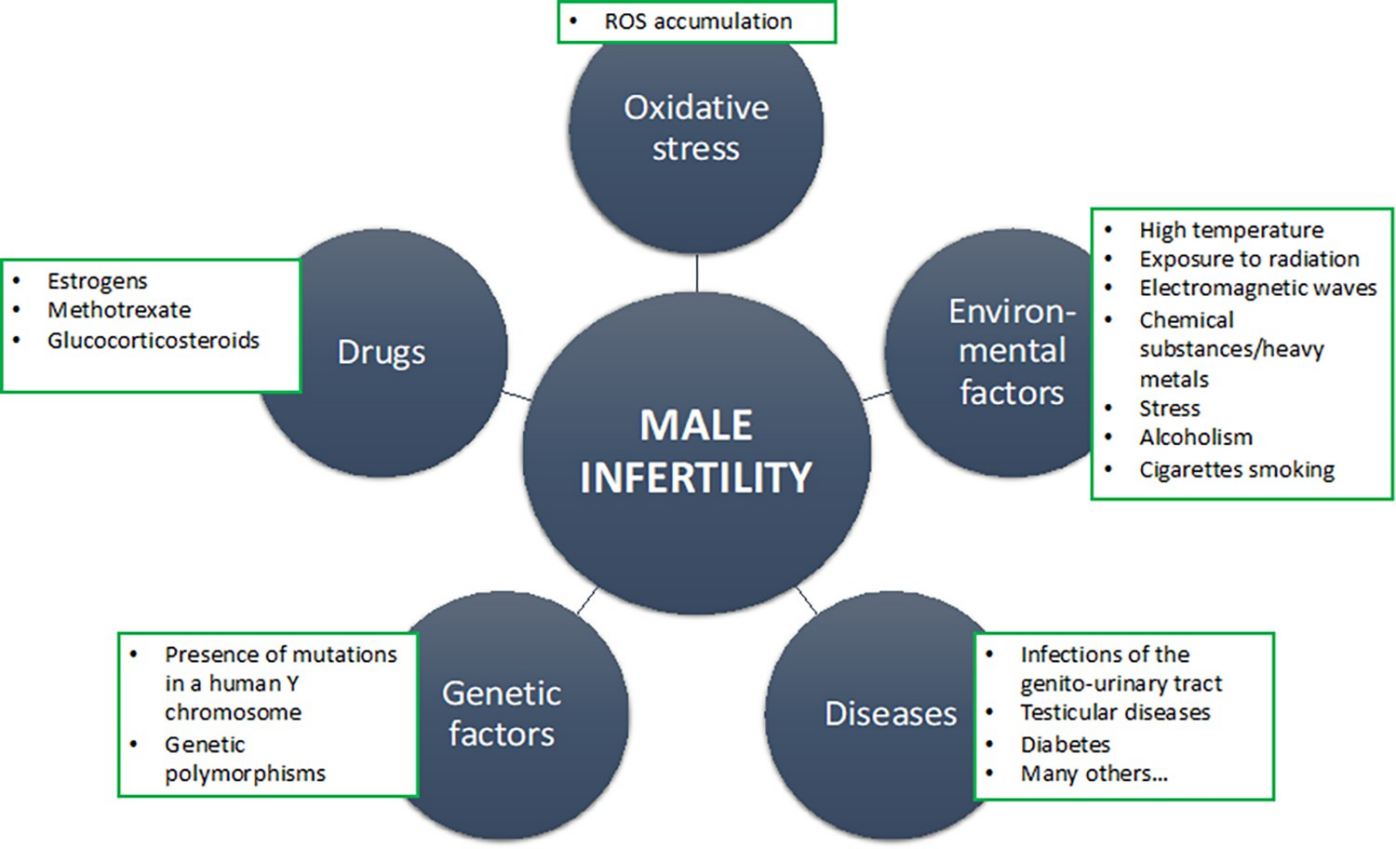
Breakdown of the main causes of decreased male fertility/infertility. Modification based on Miyamoto et al. [8] and Olaemi [9]. ROS–Reactive oxygen species.

Infertility is a worldwide social problem that affects many couples who would like to have offspring. The definition of infertility by the World Health Organization (WHO) is as follows: inability to achieve pregnancy after one year of unprotected intercourse [1]. Currently, this crucial issue touches 48.5 million pairs globally (15% of all couples around the world) [2]. The male factor is responsible for approximately 50% of infertility cases overall (the sole male factor is 20%, and together with the female factor next 30%) [2]. Numerous systemic disorders can exert detrimental effects on male fertility, primarily manifesting as a decline in sperm quality (Fig 1).

The primary diagnostic tool for male infertility is semen analysis (spermiogram) which allows for distinguishing several types of disorders according to parameters such as total spermatozoa number, morphology, vitality, progressive motility, etc. [3, 4]. For instance, it may be teratozoospermia (abnormal sperm morphology), asthenozoospermia (reduced sperm motility), oligozoospermia (low sperm count), or mixed disorders [4]. As the spermiogram does not provide comprehensive information regarding the underlying causes of specific disorders the recent investigations are directed toward understanding infertility stemming from fluctuations in levels of molecules such as essential energy substrates for spermatozoa (e.g., sugars and lipids) [5, 6]. Therefore, it’s important to highlight seminal plasma’s role as a complex mixture containing various compounds (e.g., inorganic ions, specific hormones, proteins, and peptides, including cytokines and enzymes, cholesterol, DNA, RNA, etc.) indispensable for the proper functioning of spermatozoa [7]. Due to its rich and diverse composition, seminal plasma might have potential not only as a diagnostic fluid but also as a source of insights into the mechanisms leading to decreased male fertility [7].

There is a thesis concerned with the role of lipids in human reproduction since cholesterol is the main substrate for the synthesis of different steroids and hormones that are shown to affect steroidogenesis in men [10, 11]. Additionally, numerous scientific reports documented a correlation between fertility disorders and lifestyle such as diet, as well as systematic diseases like diabetes, which directly influence serum lipid levels (e.g., high-density lipoproteins (HDL), low-density lipoproteins (LDL) or triglycerides (TG)), carbohydrates (e.g., glucose, fructose) and nonesterified fatty acids (NEFA) [6, 9, 12]. This suggests a potential translation of the levels of various parameters in blood serum into their levels in seminal plasma.

Another significant parameter associated with fertility appears to be 5′AMP-activated protein kinase (AMPK). This enzyme serves as the primary regulator of energy homeostasis, activated by cellular low-energy states [13, 14]. Under conditions of a lack of energy, AMPK activation leads to inhibition of ATP-consuming pathways (e.g., fatty acids synthesis, cholesterol synthesis, and gluconeogenesis) and stimulation of ATP production processes (e.g., fatty acids oxidation and glycolysis) [15]. Research on mammals, including humans, has demonstrated the involvement of the AMPK activation pathway in regulating male gonadal function, semen production, and semen quality, thereby influencing male reproductive capacity [16].

Recent studies on the nature of the effects of carnitine on the human body also suggest its association with male fertility [17]. Carnitine is responsible for the transport of long-chain fatty acids, which are transferred to the mitochondria where they change, resulting in the production of energy necessary for the proper functioning of the body’s cells [17]. Interestingly, the male reproductive system exhibits a high concentration of this organic compound, particularly notable in the epididymis which appears to be related to the energy metabolism of spermatozoa and consequently to male fertility [18]. As spermatozoa in the epididymis possess the capacity to utilize fatty acids as an energy source, carnitine likely functions as a cofactor for their transport and oxidation [18]. Additionally, several studies have indicated a beneficial impact of carnitine treatment on semen parameters, particularly on sperm motility [19–21].

Sirtuin 1 (SIRT1) is also an intriguing research topic in male infertility. This molecule exhibits high expression in mammalian testicular tissue and is believed to play a crucial role in spermatogenesis [22]. Furthermore, SIRT1 action is connected with the aforementioned AMPK pathway, as simultaneous activation of SIRT1 activity and the AMPK pathway has been associated with improved sperm motility [22].

Based on the above associations, our research aimed to investigate parameters linked to spermatozoa’s energy metabolism. Specifically, our objectives were to: 1) check whether there were any differences in the levels of the analyzed seminal plasma parameters between groups of fertile and infertile men; 2) assess the correlations between parameters characterizing the quality of semen (sperm concentration, count per ejaculate, volume of ejaculate, sperm motility, progressive motility, and vitality) and 9 parameters that act as energy substrates for spermatozoa or are closely related to seminal plasma energy homeostasis in both fertile and infertile men; and 3) identify the relationships between the levels of the 9 examined parameters in seminal plasma and serum in both fertile and infertile men. Additionally, we intended to quantify SIRT1 concentrations in the seminal plasma and serum from all participants.

## Materials and methods

### Study design

The study was designed as a cross-sectional investigation involving Polish men aged 20–50. The minimum sample size was determined based on an α error probability of 0.5, a power of 0.8, and a minimum effect size of 0.4, yielding a required sample size of 34 patients. However, only 25 fertile volunteers could be recruited for the study at the time of data collection. Post hoc power analysis for the correlation test, assuming a minimum effect size of r = 0.4, an α error probability of 0.05, and a sample size of 25, yielded a power of 0.68. These calculations were performed using the online G*Power calculator (v. 3.1.9.6, developed by Franz Faul, Universitas Kiel, Germany, https://www.psychologie.hhu.de/arbeitsgruppen/allgemeine-psychologie-und-arbeitspsychologie/gpower).

### Patients

The seminal plasmas and blood sera were collected from 88 infertile male patients visiting the Clinical Centre of Gynaecology, Obstetrics, and Neonatology in Opole (Poland) based on the consent of the Bioethics Committee of Wroclaw Medical University No. KB-549/2019 of July 01, 2019. The infertile participants’ recruitment period started on July 14, 2019, and ended on June 30, 2021. The seminal plasma and blood sera from 25 men with proven fertility were collected in 2021 among volunteers who visited the Department of Laboratory Diagnostics, Wroclaw Medical University (Poland) based on the consent of the Bioethics Committee of Wroclaw Medical University No. KB-30/2021 of January 19, 2021. All participants of the study were informed about its aims and were given instructions about their rights. All patients gave conscious written permission for using their biological material for research purposes and the study was conducted according to the guidelines of the Second Declaration of Helsinki. The inclusion criteria for the study were the willingness to participate in the study and proper preparation for blood and semen donation. However, active and/or past conditions such as cancer, mumps orchitis, adenoma, cardiovascular diseases, nephritis, hepatitis, diabetes, eating disorders, inflammation in the genitourinary system, Klinefelter’s syndrome, cryptorchidism, torsion of the testicle, varicocele, sexually transmitted diseases, obesity, neurological disorders, tuberculosis, history of injury, damage and/or surgery in the genitourinary system, scrotum, and groin area as well as current acute infectious diseases with high temperature were applied as exclusion criteria.

### Sampling and samples preparation

Patients’ venous blood was drawn under fasting conditions on the day the semen samples were donated. After receiving the ejaculate samples (obtained by masturbation after 3–5 days of sexual abstinence), all of them were liquified at 37°C (maximum 60 min.). Then, for all of the semen samples (from fertile and infertile men), a standard analysis according to the WHO 2010 guidelines was performed [23] (i.e., semen volume, pH, and sperm viability) and supplemented by using computer-assisted sperm analysis (total sperm count in ejaculate, sperm concentration, total motility, progressive motility, and morphology), SCA Motility and Concentration, software version 6.5.0.5. (Microptic SL, Barcelona, Spain). Based on an interview collected from patients and the results of standard semen analysis, samples from participants of the study were divided into two main groups: fertile and infertile. The fertile normozoospermic group consists of volunteers with normal semen parameters and fertility confirmed by having offspring(s) not older than 2 years. The infertility group included patients who visited the clinic for infertility treatment and had semen disorders such as teratozoospermia (T, n = 32), asthenoteratozoospermia (AT, n = 30), and oligoasthenoteratozoospermia (OAT, n = 26). Due to the release of new WHO guidelines in 2021 [4] (biological sample collection took place before this year), patients were reclassified into the appropriate groups of infertile men according to the latest WHO recommendations before the commencement of the experiment. Semen samples with leukocytospermia and with the presence of bacteria in the microscopic examination were not included in the study. Ejaculates were centrifuged at 3500×g for 10 minutes at room temperature, while clotted blood samples were centrifuged for 10 minutes at 2000×g at room temperature to obtain seminal plasmas and blood sera, respectively. Then, both seminal plasma and blood sera were divided into aliquots and frozen at –86°C until examination.

### Assay measurements

All parameters were determined both in seminal plasmas and blood sera, except fructose concentration, which was measured only in seminal plasmas. The analyses were performed according to the manufacturer’s instructions, and measurements were made in undiluted seminal plasmas and blood sera. Detailed information on applied biochemical laboratory methods for glucose, total cholesterol, high-density lipoprotein cholesterol, low-density lipoprotein cholesterol, triglycerides, and non-esterified fatty acids examinations is attached as S1 Table. All those parameters were determined using a Konelab 20i biochemical analyzer (Thermo Scientific, Vantaa, Finland). AMPK concentrations were determined with a Human (AMPK) ELISA Kit (SunredBio, China) and SIRT1 with ELISA kit (Genorise Scientific, Inc. USA,). To assess the carnitine concentrations, the colorimetric method based on the transfer of the acetyl group from coenzyme A to carnitine was used (Abcam, UK). Fructose concentrations were determined by the photometric test based on the colored complex production with indole (FertiPro NV, Belgium). AMPK, carnitine, and fructose levels were evaluated using a Mindray-96A microplate reader (Mindray, Shenzhen, China).

### Statistical analysis

Statistica 13.3PL software (StatSoft Poland Sp. z o.o., Krakow, Poland) was used to analyse the data. The Shapiro-Wilk tests were employed to assess the adherence of parameters to a normal distribution. Most of the seminal plasma and blood serum parameters exhibited deviation from the normal distribution, thus necessitating the application of nonparametric statistical tests for all analyses. The results are presented as the median (Me) with the interquartile range (Q1–Q3). Differences in seminal plasma and blood serum parameters between fertile and infertile groups were evaluated using the Mann-Whitney U test. Spearman’s rank correlation was used to evaluate the associations between examined seminal plasma parameters and semen parameters (sperm concentration, count per ejaculate, volume of ejaculate, sperm motility, progressive motility of sperm, and vitality of sperm) in both fertile and infertile groups of men. The Spearman’s rank correlation was also checked between both biological materials tested, seminal plasmas and blood sera, in concentrations of determined parameters. The strength of Spearman’s rank correlations in all analyses was rated based on the following classification: 0.0 ≤ |R| ≤ 0.2—lack of correlation; 0.2 < |R| ≤ 0.4—weak correlation; 0.4 < |R| ≤ 0.7—moderate correlation; 0.7 < |R| ≤ 0.9—strong correlation; and 0.9 < |R| ≤ 1.0—very strong correlation. A two-tailed p-value of less than 0.05 was considered significant. The research data file is fully available from 24/01/2024.

## Results

### Comparison of the examined parameters between fertile and infertile men

After the determination of the concentration of 9 parameters in all seminal plasmas and blood sera: their values were compared between fertile men (N = 25) with proven fertility and infertile patients (N = 88) with abnormal semen parameters. The obtained results are shown in Table 1.

**Table 1:** The seminal plasma values of analyzed parameters in the groups of fertile and infertile men.

	Seminal Plasma			Blood serum		
Group Parameter	Fertile (*N =* 25) Me (Q1–Q3)	Infertile (*N* = 88) Me (Q1–Q3)	p	Fertile (*N =* 25) Me (Q1–Q3)	Infertile (*N* = 88) Me (Q1–Q3)	p
Glucose [mmol/L]	0.056 (0.056–0.122)	0.112 (0.056–0.112)	**0.035**	5.12 (5.06–5.43)	5.10 (4.68–5.52)	0.389
Total cholesterol [mmol/L]	0.517 (0.233–0.672)	0.642 (0.349–0.956)	0.093	5.14 (4.60–5.56)	5.72 (5.27–6.91)	**0.002**
HDL [mmol/L]	0.060 (0.052–0.078)	0.052 (0.052–0.052)	**< 0.001**	1.37 (1.26–1.42)	1.30 (1.11–1.48)	0.327
LDL [mmol/L]	0.439 (0.284–0.698)	0.478 (0.297–0.620)	0.795	2.76 (2.40–3.20)	3.06 (2.52–3.31)	0.300
TG [mmol/L]	0.284 (0.129–0.887)	0.555 (0.284–0.956)	0.552	2.42 (1.78–3.35)	2.67 (2.00–4.02)	0.552
NEFA [mmol/L]	0.073 (0.048–0.104)	0.087 (0.050–0.116)	0.264	0.497 (0.437–0.627)	0.450 (0.307–0.621)	0.264
AMPK [ng/ml]	21.7 (17.6–25.1)	22.4 (17.6–27.1)	0.515	32.3 (20.0–92.3)	38.1 (20.8–107.9)	0.826
Carnitine [mM/L]	15.7 (11.5–22.5)	14.3 (8.97–16.9)	0.105	9.88 (8.75–10.88)	8.86 (7.03–11.07)	0.590
Fructose [mg/dL]	2.45 (1.97–2.76)	2.99 (2.37–3.80)	**0.045**	nd.	nd.	

A two-tailed p-value of less than 0.05 was considered significant and bolded. HDL–high-density lipoproteins; LDL–low-density lipoproteins; TG–triglycerides; NEFA–nonesterified fatty acid; AMPK–AMP-activated protein kinase; N–number of participants; Me–median, nd.–nondetermined.

We observed significant differences in seminal plasma glucose, HDL, and fructose concentrations between groups of fertile and infertile men. Glucose and fructose concentrations were significantly higher in the group of infertile men, while HDL concentrations were significantly lower in the abovementioned group when compared with the group of fertile men. There were no significant differences in the values of other examined seminal plasma parameters between the groups of participants. For serum samples, significant differences were found only for cholesterol concentration, which was higher in the group of infertile men.

### Correlations between biochemical and energetic parameters and semen parameters

To determine the correlations between seminal plasma parameters and semen parameters (sperm concentration and count per ejaculate, the volume of ejaculate, sperm motility, progressive motility, and vitality), both in fertile and infertile groups of participants, Spearman’s rank correlation was used. All the results obtained are presented in Table 2.

**Table 2:** Correlations between semen parameters and examined seminal plasmas constituents in fertile and infertile men.

**Fertile men n = 25**							
**Parameter**	**Correlation coefficient (r)** ***p-value***	**Concentration**	**Count per ejaculate**	**Volume**	**Motility**	**Progressive motility**	**Vitality**
**Glucose**	r	–0.292	**–0.423**	–0.292	–0.379	–0.181	–0.051
	*p*	*0*.*156*	***0*.*035***	*0*.*178*	*0*.*062*	*0*.*385*	*0*.*808*
**Total Cholesterol**	r	–0.110	–0.265	–0.241	**–0.425**	–0.258	–0.227
	*p*	*0*.*601*	*0*.*200*	*0*.*245*	***0*.*034***	*0*.*214*	*0*.*276*
**HDL**	r	0.158	0.071	–0.108	–0.095	0.007	0.160
	*p*	*0*.*450*	*0*.*737*	*0*.*606*	*0*.*651*	*0*.*972*	*0*.*444*
**LDL**	r	–0.104	–0.218	–0.248	**–0.466**	–0.351	–0.390
	*p*	*0*.*622*	*0*.*295*	*0*.*231*	***0*.*019***	*0*.*085*	*0*.*054*
**TG**	r	**–0.397**	**–0.428**	–0.201	**–0.542**	–0.328	**–0.619**
	*p*	***0*.*049***	***0*.*033***	*0*.*335*	***0*.*005***	*0*.*110*	***0*.*001***
**NEFA**	r	–0.016	–0.141	–0.368	–0.384	0.328	–0.355
	*p*	*0*.*940*	*0*.*502*	*0*.*070*	*0*.*058*	*0*.*110*	*0*.*082*
**AMPK**	r	0.003	–0.094	0.137	–0.084	**–0.481**	–0.125
	*p*	*0*.*990*	*0*.*711*	*0*.*587*	*0*.*742*	***0*.*043***	*0*.*622*
**Carnitine**	r	0.040	0.118	–0.088	0.153	0.435	0.232
	*p*	*0*.*874*	*0*.*642*	*0*.*727*	*0*.*545*	*0*.*071*	*0*.*354*
**Fructose**	r	–0.038	–0.190	–0.343	–0.299	–0.098	–0.043
	*p*	*0*.*870*	*0*.*410*	*0*.*127*	*0*.*187*	*0*.*672*	*0*.*852*
**Infertile men n = 88**							
**Parameter**	**Correlation coefficient (r)** ***p-value***	**Concentration**	**Count per ejaculate**	**Volume**	**Motility**	**Progressive motility**	**Vitality**
**Glucose**	r	0.012	–0.048	–0.096	0.025	0.025	0.029
	*p*	*0*.*910*	*0*.*658*	*0*.*373*	*0*.*815*	*0*.*820*	*0*.*787*
**Total Cholesterol**	r	0.185	0.147	–0.012	0.003	0.004	0.000
	*p*	*0*.*084*	*0*.*172*	*0*.*911*	*0*.*979*	*0*.*973*	*0*.*998*
**HDL**	r	**0.217**	0.205	0.005	0.002	–0.008	0.004
	*p*	***0*.*042***	*0*.*056*	*0*.*962*	*0*.*988*	*0*.*938*	*0*.*973*
**LDL**	r	**0.211**	0.162	–0.015	0.059	0.042	0.050
	*p*	***0*.*048***	*0*.*133*	*0*.*887*	*0*.*584*	*0*.*706*	*0*.*644*
**TG**	r	**–0.222**	**–0.285**	**–0.237**	–0.177	–0.163	–0.184
	*p*	***0*.*037***	***0*.*007***	***0*.*026***	*0*.*098*	*0*.*128*	*0*.*087*
**NEFA**	r	0.106	0.056	–0.139	–0.097	–0.061	–0.086
	*p*	*0*.*324*	*0*.*606*	*0*.*195*	*0*.*369*	*0*.*575*	*0*.*428*
**AMPK**	r	–0.098	–0.091	0.063	**–0.254**	**–0.276**	**–0.237**
	*p*	*0*.*370*	*0*.*406*	*0*.*568*	***0*.*019***	***0*.*010***	***0*.*029***
**Carnitine**	r	–0.028	0.013	–0.006	0.012	0.037	–0.013
	*p*	*0*.*796*	*0*.*906*	*0*.*956*	*0*.*911*	*0*.*731*	*0*.*905*
**Fructose**	r	–0.196	–0.190	**0.337**	–0.120	**–0.232**	–0.127
	*p*	*0*.*089*	*0*.*410*	***0*.*003***	*0*.*301*	***0*.*044***	*0*.*276*

Two-tailed *p-*values of less than 0.05 were considered significant and bolded. r–Spearman’s rank values. HDL–high-density lipoprotein; LDL–low-density lipoprotein; TG–triglycerides; NEFA–non-esterified fatty acids; AMPK–AMP-activated protein kinase.

#### Sperm concentration

In both groups of fertile and infertile men, weak negative correlations between TG levels and sperm concentrations were observed. Additionally, in the group of infertile men, positive weak correlations with sperm concentration were observed for HDL and LDL concentrations.

#### Sperm count in ejaculate

In a group of fertile men, we observed moderate negative correlations between seminal plasma glucose and TG concentrations and sperm count in the ejaculate as well as weak negative correlations between TG concentrations and sperm count in the seminal plasma of infertile men.

#### Volume of ejaculate

The volume of ejaculate was weakly negatively correlated with TG and fructose concentrations only in the group of infertile patients. No correlations were observed between the volume of ejaculate and the determined seminal plasma parameters in fertile men.

#### Sperm motility and progressive motility

Our study shows that the concentrations of parameters such as total cholesterol, LDL, and TG are moderately negatively correlated with sperm motility in the group of fertile men. A moderate negative correlation was also observed between the progressive motility of spermatozoa and AMPK levels in fertile men. In turn, for a group of infertile men, there was a weak negative correlation between sperm motility and AMPK levels. Similar associations were observed between the progressive motility of spermatozoa and AMPK levels in infertile patients, but negative weak correlations with fructose concentrations were also present.

#### Sperm vitality

The highest Spearman’s rank values were observed in the group of fertile men between sperm vitality and determined seminal plasma parameters. Although these correlations were moderate, it was observed that sperm vitality was negatively correlated with TG concentrations. On the other hand, in infertile male patients, we observed weak negative correlations between this semen parameter and AMPK concentrations.

**Fig 2:**
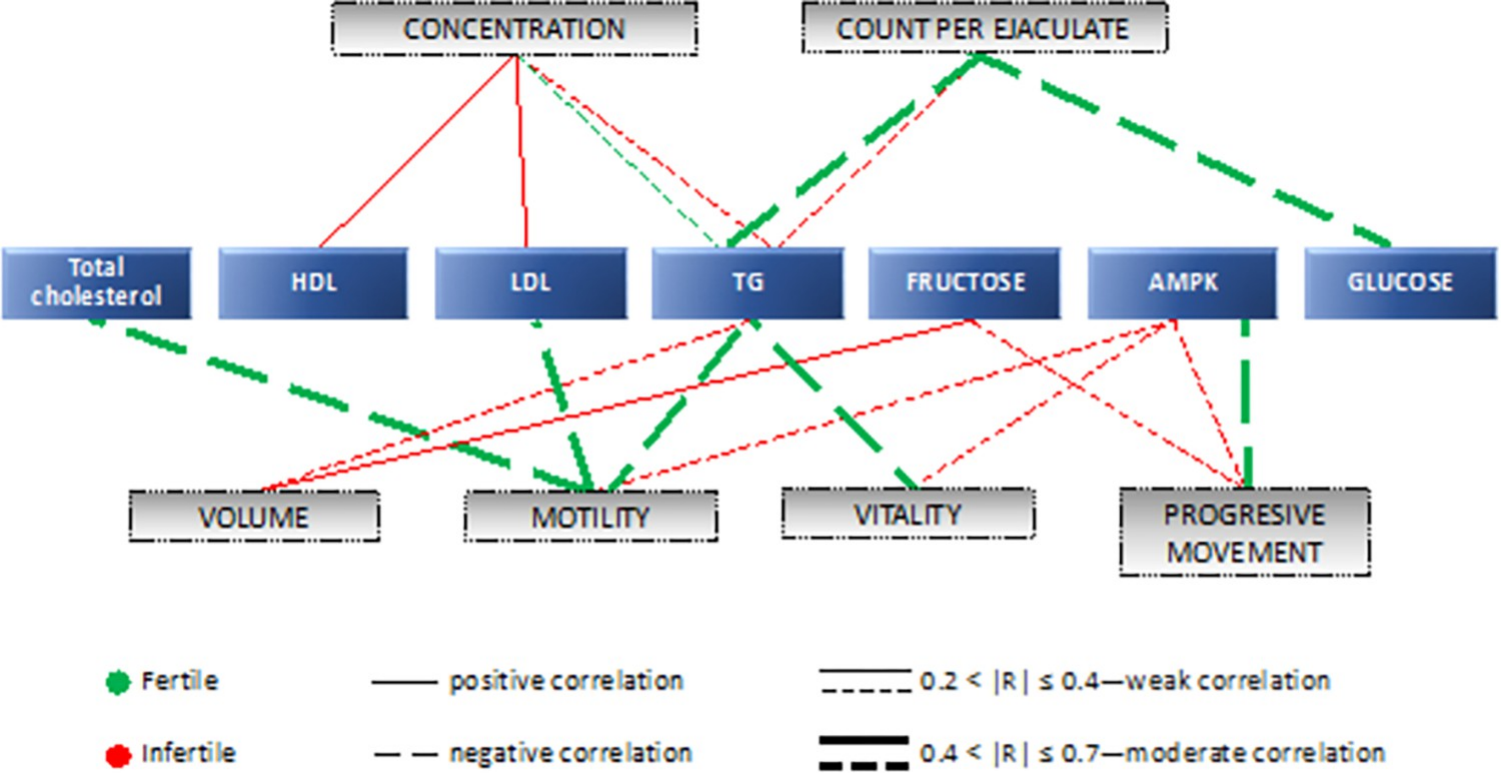
Graphical representation of significant correlations between semen parameters and examined seminal plasma parameters. HDL–high-density lipoproteins; LDL–low-density lipoproteins; TG–triglycerides; AMPK–AMP-activated protein kinase.

Fig 2 shows all significant correlations between seminal plasma parameters determined in this study and ejaculate parameters for both studied groups of men.

### Correlations between blood serum and seminal plasma parameters

The concentrations of all 9 parameters were also determined in the blood sera and then the analysis of Spearman’s rank correlations was used to assess the associations between measured parameters separately for each studied group (Table 3). The strength of Spearman’s rank correlations was rated based on the classification mentioned in the Materials and Methods section. We observed the presence of positive correlations between blood sera and seminal plasmas in concentrations of three examined parameters: LDL in the fertile group of men (moderate correlation), AMPK, and carnitine (weak correlation) in the infertile patients.

**Table 3:** Correlations between blood serum and seminal plasma parameters.

Parameter	Correlation coefficient (r) *p-value*	Fertile n = 25	Infertile n = 88
Glucose	r	0.061	0.085
	*p*	*0*.*773*	*0*.*430*
Total cholesterol	r	0.355	0.177
	*p*	*0*.*082*	*0*.*099*
HDL	r	–0.106	–0.079
	*p*	*0*.*613*	*0*.*460*
LDL	r	**0.579**	0.146
	*p*	***0*.*002***	*0*.*174*
TG	r	0.176	0.131
	*p*	*0*.*400*	*0*.*223*
NEFA	r	–0.339	–0.026
	*p*	*0*.*097*	*0*.*812*
AMPK	r	0.118	**0.293**
	*p*	*0*.*642*	***0*.*006***
Carnitine	r	–0.051	**0.379**
	*p*	*0*.*840*	***< 0*.*001***

A two-tailed *p-value* of less than 0.05 was considered significant and bolded. r–Spearman’s rank values. HDL–high-density lipoproteins; LDL–low-density lipoproteins; TG–triglycerides; NEFA–nonesterified fatty acids; AMPK–AMP-activated protein kinase.

### SIRT1 in the seminal plasmas and blood sera

The additional parameter examined in our study was SIRT1 in the seminal plasmas and blood sera of fertile and infertile men. The concentration determinations of this enzyme were impossible in seminal plasmas, as SIRT1 was undetectable in this biological material in both groups of participants. In the blood sera, it was only possible to detect SIRT1 in the range of 0.04–24.50 ng/ml in 20% of all sera (5 out of 25 samples) derived from fertile men and in 30.7% of sera (27 out of 88 samples) of infertile men.

## Discussion

The role of seminal plasma in each of the processes leading to fertilization has turned out to be crucial in recent years for male fertility and the fertilization process. The enormous amount of molecules and compounds contained in it has led scientists from around the world to emphasize their importance and to try to identify the relationships between semen parameters (and, consequently, between widely understood fertility) and biochemical parameters [7]. However, the role of various components contained in human seminal plasma is still not well known.

The analysis conducted by us showed significant differences in the levels of 3 of 9 determined parameters between the seminal plasma of the studied fertile and infertile men; we perceived that only glucose, HDL, and fructose levels differed significantly. The objective of this study was also to investigate the correlations between seminal plasma parameters associated with energy metabolism and semen parameters in both fertile and infertile men. An additional aspect of our research was to analyze the correlations between biochemical parameters present in both blood serum and seminal plasma. Discrepancies in findings across studies conducted by various authors examining such correlations are likely attributable to the diverse array of disorders contributing to male infertility, as well as variations in lifestyle, diet, origin, demographic factors such as age and race, and other individual characteristics. While correlations between seminal biochemical parameters and semen quality in fertile men are relatively scarce in the literature, there is a greater emphasis on investigating such associations in infertile men. However, this aspect of research is often limited to specific populations. To ensure meaningful comparisons, this discussion exclusively considers reports about humans.

We hypothesized that the significantly higher levels of seminal plasma glucose in infertile patients observed by us are most likely a result of male infertility rather than its cause. This is supported by the fact that spermatozoa utilize glucose as an energy substrate through membrane transporters (e.g., GLUT), transporting glucose from the external environment to the interior and using it in metabolic processes [24, 25]. Due to factors like decreased sperm concentration and vitality often observed in male infertility [4], spermatozoa utilize less glucose, resulting in higher levels of glucose remaining in the seminal plasma. Moreover, Mehrparvar et al. [26] found higher glucose concentration in seminal plasma among patients with teratozoospermia compared to the control group. They suggested that the increase might result from reduced glucose utilization by the greater number of malformed spermatozoa. Additionally, Martikainen et al. [27] observed that in the group of infertile men, seminal glucose levels were higher (0.41 ± 0.26 mmol/L) than in fertile men (0.24 ± 0.13 mmol/L); however, they examined semen instead of seminal plasma, which explain why the glucose concentrations were higher than we observed in seminal plasma.

We identified negative correlations between sperm count and seminal plasma glucose concentrations only among fertile men, while Zhang et al. [28] documented similar negative correlations only among infertile men. It is essential to underscore the disparities between our investigations and those conducted by Zhang et al. [28], notably pertaining to demographic variations, as our study concentrated on subjects from Poland, whereas Zhang et al. [28] examined a Chinese population.

Lipids present in seminal plasma are important for reproduction processes, as documented by numerous authors. Davis et al. [29, 30] observed a decrease in sperm cell membrane cholesterol levels during capacitation, leading to modification of lipid composition and an imbalance between cholesterol and phospholipid levels. Mehrparvar et al. [26] also observed associations between seminal plasma cholesterol levels and male fertility. The authors documented that in teratozoospermic men, the seminal plasma total cholesterol levels were lower than those observed in normozoospermic fertile men. However, our study found no significant differences in cholesterol concentrations between seminal plasmas of fertile and infertile men (p > 0.05). Valsa et al. [31] and Calonge et al. [32] also reported that seminal plasma cholesterol levels are similar for fertile and infertile men.

Although seminal plasma total cholesterol concentrations did not significantly differ between the examined groups, infertile patients exhibited notably lower levels of seminal plasma HDL. In contrast to previous findings by Lu et al. [33], our study revealed a positive correlation between HDL levels and sperm concentration. Additionally, in our study, no significant differences were observed between fertile and infertile men in seminal plasma LDL concentrations. This also contradicts the findings of Lu et al. [33], who reported higher seminal plasma LDL levels in the infertile group. Such discrepancies highlight the importance of geographical region, dietary habits, and lifestyle differences in male fertility and their potential impact on biochemical parameters.

Triglycerides, another parameter closely related to lipid metabolism, were observed at higher levels in seminal plasmas of infertile patients examined by Calonge et al. [32]. Our findings diverge from those of Calonge et al. [32], as we did not observe significant differences in TG levels between fertile and infertile men. However, it is noteworthy that TG levels appeared visibly higher in the infertile group. A possible explanation for the discrepancy in the results obtained by us and by Calonge et al. [32] may be that the authors examined the seminal plasmas of oligoasthenozoospermic men, and we did not have such a group of patients. In this case, Lu et al. [33] also observed that higher concentrations of triglycerides are present in the seminal plasma of infertile men compared to fertile counterparts.

We showed several correlations between the levels of lipid and sperm parameters in both fertile and infertile men. In fertile men, there was a negative correlation between sperm concentration and seminal plasma concentrations of triglycerides. In infertile men, a positive correlation was found between sperm concentration and seminal plasma HDL and LDL levels, while a negative correlation with both sperm concentration and sperm count was noted with TG concentrations. In contrast, Lu et al. [33] showed negative correlations between sperm concentration and seminal plasma HDL levels and no correlations concerning seminal plasma LDL and TG concentrations in infertile men. Additionally, both Lu et al. [33] and Zhang et al. [28] did not observe correlations between seminal plasma TG concentrations and sperm count in infertile men. In the present study, we also observed negative correlations between semen volume and seminal plasma TG concentrations for infertile men which is also opposite to the results obtained by Zhang et al. [28], who observed a positive correlation in this regard. All of these disparities may stem from the diverse origins of the participants, as our study involved Polish men, whereas Lu et al. [33] and Zhang et al. [28] examined individuals from China. Notwithstanding the contradictions, it seems correct to say that the parameters associated with the seminal plasma lipid profile are associated with the concentration of spermatozoa, but the direction of these relationships may vary depending on additional individual factors. To the best of our knowledge, we are the first to show significant correlations between TG and LDL levels and sperm motility in fertile men as well as negative correlations between sperm vitality and seminal plasma TG in infertile men.

Some authors have suggested that the lipids in the seminal plasma do not necessarily come from the blood but can also be produced by epithelial cells in the male reproductive tract [34]. However, we found a positive correlation between serum LDL levels and seminal plasma LDL levels among fertile men. In the group of infertile patients, we observed a negative correlation between serum and seminal plasma HDL concentrations and a lack of correlations between other parameters associated with the body’s lipid metabolism (total cholesterol, LDL, triglycerides). Our observations are similar to the results obtained by Lu et al. [33], who showed no correlations between the concentrations of some lipids (total cholesterol, LDL) between blood sera and seminal plasmas of infertile men, but in contrast to our results, they observed positive correlations in TG levels and no correlations in HDL levels between both biological materials. As we noticed before, the place of life and origin of men together with dietary habits and lifestyle may be crucial in such types of investigations and may influence the results.

In 1989, Vignon et al. [35] examined the level of nonesterified fatty acids in semen and seminal plasma after sufficiently long incubation and reported that this parameter is a source of energy for sperm. Calonge et al. [32] also examined nonesterified fatty acids and observed higher seminal plasma NEFA levels in infertile patients. Additionally, in this case, differences observed by us are insignificant, but slightly higher concentrations of this parameter in the infertile group of men are visible.

The latest reports suggest a relationship between AMPK phosphorylation and sperm capacitation [36]. Notwithstanding the lack of significant differences between fertile and infertile men in seminal plasma AMPK concentrations in our study, there is scientific evidence suggesting an effect of higher seminal plasma AMPK concentration on male fertility. Cao et al. [37] reported that oxidative stress influences AMPK activation and causes a decrease in sperm activity and stability, which ultimately leads to asthenozoospermia.

In our study, we observed negative correlations between sperm motility and AMPK concentrations in infertile men. Conversely, Calle-Guisado et al. [38] reported a decrease in AMPK concentration in human spermatozoa leading to a significant reduction in sperm motility and speed. The mechanism of such changes is not known to us, but we believe that in the case of studies of AMPK concentrations in the context of semen parameters, it is important to emphasize whether the examined biological material is full ejaculate or seminal plasma. The reason for that is that AMPK concentrations depend on the energy status of the sperm cell, which is also linked with the transformation of energy substrates for spermatozoa, present in the semen microenvironment, into derivatives inaccessible for spermatozoa. This may explain the negative correlations between the concentrations of this enzyme and the spermatozoa’s ability to move. Additionally, negative correlations were also observed between the progressive motility and seminal plasma AMPK concentrations in both fertile and infertile men as well as between sperm vitality and seminal plasma AMPK concentration in infertile patients. To the best of our knowledge, similar correlations between these parameters have not been demonstrated by other authors.

Similarly, the correlations between seminal plasma and blood serum AMPK concentrations remain unexplored. We identified positive correlations between levels of this parameter in sera and seminal plasmas in infertile men. Oxidative stress may contribute to changes in AMPK activation, which in turn influences male fertility [37]. Therefore, we can postulate that the influence of oxidative stress on the human body translates into changes in the functioning of many spheres of the body at the same time and that the changes in blood serum AMPK activation may translate into changes in the levels of seminal plasma AMPK.

It was documented that carnitine is released from the epididymis into the seminal fluid, which suggests that it is not related to the spermatogenesis that takes place in the seminiferous epithelium [39]. As a low-molecular-weight antioxidant, carnitine is related to the prevention of changes in semen triggered by oxidative stress (including lipid peroxidation) [40]. Carnitine acts as a carrier for long-chain fatty acids which are an energy substrate for spermatozoa. Studies by Menchini-Fabris et al. [41] and Matalliotakis et al. [42] demonstrated higher seminal plasma carnitine concentrations in fertile men compared to infertile patients, particularly those with azoospermia. Although our study also noted slightly elevated carnitine levels in fertile men, the differences between the groups were not significant.

It is well known that seminal plasma carnitine is of blood origin and is collected in the epididymal lumen, where it is absorbed by spermatozoa [39]. In our study, we observed positive correlations in the levels of carnitine between blood sera and seminal plasmas in infertile men. However, we did not observe associations between carnitine levels and semen parameters.

Apart from glucose, fructose is the main carbohydrate energy substrate for spermatozoa. Consequently, variations in seminal plasma fructose concentrations are expected to differ depending on the male fertility potential. We showed that infertile men have a higher level of seminal plasma fructose. Our findings are in accordance with the results obtained by Toragall et al. [12], who demonstrated that fructose concentration decreases as the sperm concentration increases and vice versa. On the other hand, Martikainen et al. [27] observed that the fructose levels in semen did not differ significantly (p > 0.05) between infertile and fertile men; however, the authors examined full semen, and throughout the duration of the study, fructose was consumed by spermatozoa, even in samples of patients with sperm dysfunction [27].

Regarding seminal plasma fructose concentrations, our findings align with those of Said et al. [43]. The authors observed positive correlations between seminal plasma fructose concentrations and semen volume in infertile men. In contrast, Shemshaki et al. [44] found no correlation between seminal plasma fructose levels and semen volume. These discrepancies may stem from variations in the demographics of the studied populations. Furthermore, among infertile men, we identified negative correlations between seminal plasma fructose concentrations and progressive motility. On the other hand, Said et al. [43] found no correlations between ejaculate fructose levels and spermatozoa’s progressive motility. These differences may arise from variations in ethnic origins (Africa vs. Europe) of the studied populations, as well as differences in sample sizes and the type of biological material analyzed (seminal plasma vs. full ejaculate).

In summary, the correlations presented in this study indicate a potential relationship between semen parameters and seminal plasma biochemical parameters. We can draw several conclusions and future research directions based on the results obtained.

Firstly, the increased carbohydrate concentrations observed in the seminal plasma of infertile patients may result from reduced sugar consumption by spermatozoa. Therefore, their increased concentrations may be a useful biomarker in the diagnostics of fertility caused by sperm parameter disorders in men.

Secondly, while the seminal plasma lipid levels are largely independent of serum levels, seminal plasma TG concentrations appear associated with semen production processes, influencing sperm concentration, count, and volume. The mechanism of their influence remains unclear, but we can suppose that an excess of these lipid components hurts spermatogenesis. However, at a later stage in spermatozoa life, TG may be an energy substrate for spermatozoa [45], which would explain the negative correlations between their seminal plasma concentrations and sperm parameters such as motility and viability.

Another interesting research direction is AMPK. The level of AMPK in the seminal plasma is probably related to that in the blood serum and is associated with male fertility through inverse relationships between its levels and disorders of parameters related to sperm viability. In the case of lowering the energy level in sperm, the concentration of AMPK seems to increase, which translates into a negative correlation of this parameter with parameters related to spermatozoa viability, motility, and progressive motility. It was reported that alterations in AMPK/mTORC2 signaling in the sperm microenvironment may result in abnormal glucose and lipid metabolism, which impairs energy production [36]. We believe that interfering with this signaling pathway by affecting the concentration of AMPK in the spermatozoa environment can bring interesting therapeutic effects manifested in increasing spermatozoa viability and motility.

Notwithstanding the strategies made up for improving the activity of SIRTs to cope with metabolic disorders and related afflictions (including obesity-induced male infertility), it is still not well known how SIRT1 influences semen quality [22]. Although our aim was also to check the associations between the concentrations of this enzyme and the parameters of semen, it was impossible due to the inability to detect SIRT1 with a commercial test chosen by us. However, we believe that SIRT1 may be a potential therapeutic target and should be widely investigated, especially since significant differences in the levels of this enzyme were demonstrated between fertile men and infertile male patients [46].

The available literature presents many conflicting conclusions. Many authors have reported different associations between seminal plasma biochemical parameters and semen parameters, which we did not observe [28, 31, 33, 39, 41, 42, 47, 48]. The undoubted cause of such differences is many individual factors that affect male fertility, such as age, lifestyle, diet, origin, region of residence, exposure to various types of pollution, and many others. The research methodology also varied between research groups. In our study, we demonstrated that in many cases, the interpretation of the results obtained depends on the biological material in which the analyzed parameters were determined. These and many other factors indicated that there is a strong need for population studies that would also consider demographic factors.

Based on our research findings, it can be concluded that seminal plasma has the potential to emerge as a valuable source of information for diagnosing and treating male infertility. However, the relatively small control group of men with proven fertility, as well as the lack of information regarding the duration of infertility, dietary habits, lifestyle factors, and comorbidities, constitute the limitations of our study. Nevertheless, according to our findings, several parameters show potential as biomarkers capable of distinguishing fertile and infertile men, and the identified correlations may serve as a starting point for elucidating the mechanisms underlying male fertility reduction, as well as for developing future preventive and therapeutic strategies in male infertility. It is also worth noting that the present study is the first to shed light on the correlation between AMPK concentration in seminal plasma and semen parameters. Moreover, we utilized the latest WHO criteria for semen processing from 2021. This allowed our experiments to potentially reflect the correlations between biochemical parameters and semen quality in a more up-to-date manner.

## Conclusions

Male infertility is a growing health and social problem worldwide. A strong need to address the decline in male fertility raises the need to discover biomarkers useful in the diagnosis of this disease, as well as those that correlate with values of semen parameters. Our study delved into biochemical and energetic parameters in seminal plasma in fertile and infertile men, aiming to discern potential associations with semen parameters and blood serum markers. The comparison between fertile and infertile men revealed significant differences in seminal plasma glucose, fructose, and HDL concentrations. Specifically, infertile men exhibited higher levels of glucose and fructose but lower levels of HDL compared to their fertile counterparts. Our analysis also unveiled associations between examined seminal plasma parameters and semen parameters. Notably, weak negative correlations between seminal plasma TG levels and sperm concentration were observed in both fertile and infertile groups, while HDL and LDL concentrations displayed positive weak correlations with sperm concentration specifically in infertile men. Additionally, seminal plasma glucose and TG concentrations exhibited moderate negative correlations with sperm count in fertile men. The semen volume displayed weak correlations with TG (negative) and fructose (positive) concentrations in infertile patients. Sperm motility was correlated with seminal plasma total cholesterol, LDL, and TG concentrations (moderate negative correlations) in fertile men and with AMPK concentrations (weak negative correlation) in seminal plasma from infertile men. Moreover, for fertile men, we observed moderate negative correlations between seminal plasma TG concentrations and sperm vitality and between progressive motility of spermatozoa and seminal plasma AMPK concentrations, whereas in infertile men both sperm vitality and progressive motility of spermatozoa were weakly negatively correlated with seminal plasma AMPK concentrations. We also observed weak negative correlations between seminal plasma fructose concentrations and progressive motility of spermatozoa. Such correlations underscore the complex interplay between metabolic factors and sperm production and parameters and highlight directions for targeted therapeutic interventions.

The analysis of correlations between blood serum and seminal plasma parameters unveiled intriguing connections, particularly regarding LDL, AMPK, and carnitine. While LDL exhibited a moderate positive correlation between blood sera and seminal plasma in fertile men, AMPK and carnitine displayed weak positive correlations in infertile patients. These findings suggest potential systemic influences on seminal plasma composition and underscore the intricate interplay between circulating factors and male reproductive health.

In conclusion, the identified correlations and disparities offer valuable directions for future research addressing male infertility challenges, which may result not only in finding biomarkers useful in male infertility diagnostics but also in new molecular targets usable in the treatment of male fertility disorders.

## Supporting information

S1 Table Detailed information on applied biochemical laboratory methods. (DOCX)
