# Supplementary material for: Exploring correlations: Human seminal plasma and blood serum biochemistry in relation to semen quality
Source: PLoS One. 2024 Jun 24;19(6):e0305861. doi: 10.1371/journal.pone.0305861 (PMC11195956; doi:10.1371/journal.pone.0305861)
Supplement: S1 Table — (DOCX) [file pone.0305861.s001.docx]

**Supporting Information**

S1Table. Detailed information on applied biochemical laboratory methods

| Parameter | Short method description | REF | Manufacturer | Intra-assay precision | Inter-assay precision |
| --- | --- | --- | --- | --- | --- |
| Glucose | This method employs glucose oxidase and a modified Trinder colour reaction, catalysed by the enzyme peroxidase. | 981780 | Thermo Scientific, Finland | <2% | <3% |
| Total cholesterol | Determination of cholesterol after enzymatic hydrolysis and followed by oxidation with cholesterol oxidase and coloured complex formation in reaction catalysed by peroxidase action | 1 1300 99 10 021 | DiaSys, Germany | <2% | <3% |
| High-density lipoprotein cholesterol | The homogeneous method with antibodies against human lipoproteins to form antigen-antibody complexes with LDL, VLDL and chylomicrons in a way that only HDL is selectively determined by an enzymatic cholesterol measurement | 1 3521 99 10 021 | DiaSys, Germany | <3% | <6% |
| Triglycerides | Colorimetric enzymatic test using glycerol-2-phosphate-oxidase followed by the coloured complex formation in reaction catalysed by peroxidase after enzymatic splitting with lipoprotein lipase | 1 5710 99 10 021 | DiaSys, Germany | <2% | <3% |
| Non-esterified fatty acids | Non-esterified fatty acid and CoA react in the presence of acetyl coenzyme A synthetase to acetylated coenzyme A, which is oxidized by acyl-coenzyme A oxidase and then coloured product is formed with reaction catalysed by peroxidase | 1 5781 99 10 935 | DiaSys, Germany | <4% | <6% |
| Low-density lipoprotein cholesterol | The homogeneous method with block polymer detergents protects human lipoproteins HDL, VLDL and chylomicrons in a way that only LDL is selectively determined by an enzymatic cholesterol measurement | 981656 | Thermo Scientific, Finland | <3% | <6% |
| Fructose | Fructose reacts, in the presence of HCl under heat, with indole and produces a coloured complex | Fructose | FertiPro NV, Belgium | <8% | <13% |
| L-Carnitine | The assay transfers an acetyl group from CoA to Carnitine and the free CoA formed is further processed with subsequent oxidation of the OxiRed probe to give an absorbance. | ab83392 | Abcam, Cambridge, UK | not determined | not determined |
| AMPK | A double-antibody sandwich enzyme-linked immunosorbent assay | 201-12-0747 | SunredBio, China | <10% | <12% |
| SIRT-1 | A double-antibody sandwich enzyme-linked immunosorbent assay | GR111450-2 | Genorise Scientific, Inc., USA | <6% | <9% |
